# Liuwei Dihuang (LWDH), a Traditional Chinese Medicinal Formula, Protects against β-Amyloid Toxicity in Transgenic *Caenorhabditis elegans*

**DOI:** 10.1371/journal.pone.0043990

**Published:** 2012-08-30

**Authors:** Jatinder S. Sangha, Xiaoli Sun, Owen S. D. Wally, Kaibin Zhang, Xiuhong Ji, Zhimin Wang, Yanwen Wang, Jeffrey Zidichouski, Balakrishnan Prithiviraj, Junzeng Zhang

**Affiliations:** 1 Department of Environmental Sciences, Nova Scotia Agricultural College, Truro, Nova Scotia, Canada; 2 Institute for Nutrisciences and Health, National Research Council Canada, Charlottetown, Prince Edward Island, Canada; 3 Institute of Chinese Materia Medica, China Academy of Chinese Medical Sciences, Beijing, People’s Republic of China; 4 School of Traditional Chinese Medicine, Capital Medical University, Beijing, People’s Republic of China; 5 Department of Biomedical Sciences, University of Prince Edward Island, Charlottetown, Prince Edward Island, Canada; 6 Department of Chemistry, University of Prince Edward Island, Charlottetown, Prince Edward Island, Canada; University of Ulster, United Kingdom

## Abstract

Liuwei Dihuang (LWDH), a classic Chinese medicinal formula, has been used to improve or restore declined functions related to aging and geriatric diseases, such as impaired mobility, vision, hearing, cognition and memory. Here, we report on the effect and possible mechanisms of LWDH mediated protection of β-amyloid (Aβ) induced paralysis in *Caenorhabditis elegans* using ethanol extract (LWDH-EE) and water extract (LWDH-WE). Chemical profiling and quantitative analysis revealed the presence of different levels of bioactive components in these extracts. LWDH-WE was rich in polar components such as monosaccharide dimers and trimers, whereas LWDH-EE was enriched in terms of phenolic compounds such as gallic acid and paeonol. *In vitro* studies revealed higher DPPH radical scavenging activity for LWDH-EE as compared to that found for LWDH-WE. Neither LWDH-EE nor LWDH-WE were effective in inhibiting aggregation of Aβ *in vitro*. By contrast, LWDH-EE effectively delayed Aβ induced paralysis in the transgenic *C. elegans* (CL4176) model which expresses human Aβ1–42. Western blot revealed no treatment induced reduction in Aβ accumulation in CL4176 although a significant reduction was observed at an early stage with respect to β-amyloid deposition in *C. elegans* strain CL2006 which constitutively expresses human Aβ1–42. In addition, LWDH-EE reduced *in vivo* reactive oxygen species (ROS) in *C. elegans* (CL4176) that correlated with increased survival of LWDH-EE treated N2 worms under juglone-induced oxidative stress. Analysis with GFP reporter strain TJ375 revealed increased expression of hsp16.2::GFP after thermal stress whereas a minute induction was observed for sod3::GFP. Quantitative gene expression analysis revealed that LWDH-EE repressed the expression of *amy1* in CL4176 while up-regulating *hsp16.2* induced by elevating temperature. Taken together, these results suggest that LWDH extracts, particularly LWDH-EE, alleviated β-amyloid induced toxicity, in part, through up-regulation of heat shock protein, antioxidant activity and reduced ROS in *C. elegans*.

## Introduction

Liuwei Dihuang (LWDH) is a classic Chinese medicinal formula that has been used for more than a thousand years in China. It is comprised of 6 Chinese herbs: Radix Rehmanniae Praeparata (prepared root of *Rehmannia glutiosa*), Fructus Corni (fruit of *Cornus officinalis*), Cortex Moutan (root bark of *Paeonia suffruticosa*), Rhizoma Dioscoreae (rhizome of *Dioscorea opposita*), Poria (scleorotia of *Poria cocos*), and Rhizoma Alismatis (rhizome of *Alisma plantago-aquatica*). LWDH is orally administered as a decoction or in the form of pills. According to the theory of traditional Chinese medicine (TCM), LWDH has the properties of tonifying the “Yin” of kidney, the fundamental system to support reproduction, development and performance over life time. LWDH has been used to improve or restore declined functions related to aging process and geriatric diseases, such as impaired mobility, vision, hearing, cognition and memory [1].

Recently, a number of studies have revealed the beneficial effects and some possible mechanisms of action of LWDH. LWDH was shown to affect the learning and memory function of senescence accelerated mice (SAM) primarily by altering the expression of a number of genes in hippocampus [2]–[5]. Serum of LWDH-treated rats had positive effect on neuronal and synaptic functions in cultured rat hippocampal neurons [6]. In addition, LWDH modulated the hypothalamus-pituitary-ovary axis in SAM model by changing the concentration of peptide neuro-mediators and estradiol receptor (ER-α) in pituitary and ovary and thus exerted the anti-aging effect [7]. Also using SAM model, Feng and Zang [8] demonstrated that LWDH decreased the concentration of interleukins (IL-2 and IL-6) in hippocampus suggesting this as one possible mechanism through which LWDH may improve learning, performance, and memory. In scopolamine (SCOP), p-chloroamphetamine (PCA), and cycloheximide (CXM)-induced amnesia rat models LWDH is believed to activate peripheral cholinergic neuronal system and modulate the central nervous system [9], [10]. More recently, in D-galactose-induced aging mouse and rat models, it was observed that LWDH treatment markedly improved the impaired learning and memory functions, possibly through restoring acetylcholine levels and cholineacetyltransferase activity, inhibiting acetylcholinesterase, and enhancing antioxidant activities in the brain [11]–[13]. In aluminum-induced dementia rat model, LWDH treatment alleviated the memory impairment by concomitantly increasing plasma SOD activity and reducing MDA levels and protected the brain from damage due to lipid peroxidation [14].

Model organisms that mimic human responses offer tremendous opportunities for testing the potential role of natural products in neurodegenerative diseases. *Caenorhabditis elegans* (Nematoda: Rhabditidae) is an advantageous, cost effective animal model as it has a short lifespan, a very well characterized genome and can be cultured with ease in the laboratory. Interestingly there is a considerable homology between the *C. elegans* and human genomes [15] and as such *C. elegans* has been successfully used as a model system to study aging and age-associated neurodegenerative diseases [16]–[18]. Transgenic *C. elegans* strains that express human β-amyloid (Aβ) has been used to understand molecular mechanisms of Aβ toxicity and also to screen for therapeutic agents aimed to ameliorate Aβ toxicity [19]–[21]. During the past decade, the utility of *C. elegans* has been eloquently used to study the health promoting effects of natural products used in TCM such as extracts from *Ginkgo biloba*, *Cinnamomum cassia* bark and *Panax ginseng* root [20], [22]–[32]. Similarly, green tea component epigallocatechin gallate [33], [34] and coffee extract [35], as well as other natural compounds like soy isoflavone [36], reserpine [37], and glaucarubinone [38] were shown to increase lifespan and stress resistance, and protect *C. elegans* against Aβ toxicity. No study, to present date, has been done on the protective effect of LWDH against Aβ in *C. elegans* model. Here we report our findings examining the effects of the major chemical constituents of water and ethanol extracts of LWDH (LWDH-WE and LWDH-EE) on antioxidant activity and Aβ aggregation. Using a transgenic *C. elegans* model of AD, we tested for the protective effects of LWDH-WE and LWDH-EE against Aβ toxicity and elucidated some of the mechanisms involved in delayed paralysis.

## Materials and Methods

### Chemicals and Reagents

1,1-Diphenyl-2-picrylhydrazyl (DPPH), Folin-Ciocalteu (FC) reagent, (+/−)-6-hydroxy-2,5,7,8-tetramethyl-chromane-2-carboxylic acid (Trolox), gallic acid, 5-hydroxymethyl furfural, paeonol, paeoniforin, ammonium acetate, formic acid, thioflavin T (ThT), glycine, 2′,7′-dichlorodihydrofluorescein diacetate (H2DCF-DA), gentamycin sulfate, 5-hydroxy-1,4-naphthoquinone (juglone), quercetin, D_2_O, methanol-d_4_, and phosphate buffered saline (PBS) were purchased from Sigma-Aldrich (St. Louis, MO, USA). Sweroside and loganin were purchased from ChromaDex (Irvine, CA, USA). Aβ1–42 was purchased from Anaspec (San Jose, CA, USA) and prepared according to reference [39]. *Ginkgo biloba* extract (EGb 761) was a kind gift from Dr. Willmar Schwabe Pharmaceuticals, Germany.

### Preparation and Chemical Profiling of Liuwei Dihuang (LWDH) Extracts

The water and ethanol (EtOH) extracts of Liuwei Dihuang were prepared from the commercial and standardized product Liuwei Dihuang concentrated pills manufactured by Henan Wanxi Pharmaceuticals Ltd. Co. (Nanyang, Henan, P. R. China). According to the Pharmacopeia of P. R. China [40], the product is made from Radix Rehmanniae Praeparata (RRP, prepared root of *Rehmannia glutiosa,* 160 g), Fructus Corni (FC, processed, fruit of *Cornus officinalis,* 80 g), Cortex Moutan (CM, root bark of *Paeonia suffruticosa,* 60 g), Rhizoma Dioscoreae (RD, rhizome of *Dioscorea opposite,* 80 g), Poria (sclerotia of *Poria cocos,* 60 g), and Rhizoma Alismatis (RA, rhizome of *Alisma plantago-aquatica,* 60 g).

The LWDH extracts were prepared as follows: the dry unpolished pills (6.9 kg) were milled and extracted twice with 95% EtOH with refluxing (30 min each, total EtOH 45 L), the solvent was then removed under reduced pressure and dried at 70°C to obtain LWDH EtOH extract (LWDH-EE, 1.12 kg). The pill powder that was left over after EtOH extraction was extracted with boiling water (30 min each, total water 60 L), the water was then removed at reduced pressure and dried at 70°C to yield LWDH water extract (LWDH-WE, 2.24 kg).

Chemical profiling of LWDH extracts was done with NMR and HPLC-MS methods. For ^1^H-NMR analysis, LWDH-EE was dissolved in methanol-*d_4_*, while LWDH-WE was dissolved in D_2_O. NMR spectra were acquired on a Bruker *Avance III* 600 MHz NMR spectrometer (Bruker Corporation, East Milton, ON) operating at 600.28 MHz ^1^H observation frequency and a temperature of 25±0.2°C. The signals were acquired, processed and analyzed using TopSpin® NMR data acquisition and processing Software (Bruker Biospin Ltd, East Milton, ON) integrated with the spectrometer.

For high performance liquid chromatography – mass spectroscopy (HPLC-MS) analysis, two types of columns were used. First, separation was conducted on an Agilent Zorbax SB-C18 RRHD (2.1×150 mm, 1.8 µm) column using Agilent HPLC 1100 with diode array detector (DAD) and mass selective detector (MSD) systems. Solvent A was water with 0.1% formic acid and solvent B was acetonitrile with 0.1% formic acid. Gradient elution started with 2% of solvent B for 5 min, and increased to 30% solvent B in 55 min and then to 100% solvent B in 20 min, with a total run time of 80 min. Column temperature was 30°C, flow rate was 0.2 mL/min. Mass spectra were obtained on Agilent MSD under the following conditions: drying gas flow (L/min): 10; nebulizer pressure (psig): 30; drying gas temperature (°C): 350; capillary voltage (V): 4000 (positive) and 3500 (negative). Separately, a Phenomenex Luna hydrophilic interaction liquid chromatography (HILIC) column (250×4.60 mm, 5 µm) was used for analysis using Agilent HPLC 1290 with 1200 series DAD and evaporative laser scattering detector (ELSD) systems. Solvent A was acetonitrile/water/50 mM ammonium acetate (pH 3.2) (50/40/10) and solvent B was acetonitrile/50 mM ammonium acetate (pH 3.2) (90/10). The gradient scheme was 0% A/100% B to 100% A/0% B in 50 min. Column temperature was 30°C, and flow rate was 0.5 mL/min. To obtain MS information, the same HPLC condition was applied for Agilent 1100 HPLC with MSD system. Parameters of MSD were the same as described above.

### Total Phenolics Measurement and Quantitative Analysis of Selected Components of LWDH Extracts

The total phenolics content of LWDH extracts was determined according to a modified Folin-Ciocalteu (FC) colorimetric method [41]. Briefly, 40 mg of each LWDH extract was dissolved in 80% MeOH with 1% acetic acid, centrifuged to remove insoluble fraction and made into 1 mL sample solution. Twenty microlitres (µL) of this extract solution was dispensed in 96-well plate. To each well, 40 µL 10% FC reagent was added, mixed and incubated for 5 min in the dark at room temperature. Then 160 µL 700 mM sodium carbonate was added, the plate was covered with Parafilm and incubated in the dark at room temperature for 1.5 h. Finally, the absorbance at 750 nm was read on a SPECTRA max M2 plate reader (Molecular Devices Corporation, CA, USA). Gallic acid was used as a standard. A five-point standard curve was plotted in a linear range from 0.025 mg/mL to 0.200 mg/mL. Total phenolics content of samples is reported as the mean±SD of gallic acid equivalents (GAE) in mg per gram of dry material (mg GAE/g) from 3 replicated measurements.

To quantify the concentrations of major components in LWDH extracts, a HPLC-DAD method was used with separation being conducted on a TSK gel ODS-100 V column (4.6×250 mm, 3 µm, Tosoh Bioscience) on Agilent HPLC 1100 system with DAD. LWDH extracts (10 mg) were accurately weighed and dissolved in 1 mL MeOH (for LWDH-EE) and water (for LWDH-WE). After 30 min of sonication the solution was filtered through 0.2 µm syringe filter and a 10 µL sample was injected into the system. For mobile phase, solvent A was water with 0.1% TFA and solvent B was acetonitrile with 0.1% TFA. Gradient was 3% B for 15 min, to 12% B at 17 min, and kept at 12% B for 16 min, then to 45% B at 39 min, with total run time of 87 min. Column temperature was set at 35°C, flow rate was 1 mL/min. The standards were accurately weighed and dissolved in methanol. Four different concentrations were used to make linear calibration curves. The linear ranges were at: 0.81 to 162.10 µg/mL for gallic acid; 0.83 to 333.30 µg/mL for 5-hydroxymethyl furfural. For sweroside, loganin, paeoniforin and paeonol, the linear range was at 0.83 to 166.67 µg/mL. The contents of these selected components in LWDH extracts with standards available from commercial sources were quantified and reported as mean±SD (mg/g) from 3 replicated analyses.

### DPPH Radical Scavenging Assay

LWDH extract solutions prepared as described in section 2.3 were used for determination of DPPH radical scavenging activity using a 96-well plate method [42] with minor modification. Briefly, 10 µL of sample solution was added in each well followed by addition of 100 µL of 761.4 µM DPPH in 80% MeOH. The samples were incubated at room temperature in the dark for 2 h and the absorbance at 515 nm was read on a SPECTRA max M2 plate reader (Molecular Devices Corporation, CA, USA). Trolox was used as a standard. A seven-point standard curve was plotted from 0.25 mg/mL to 0.88 mg/mL. The results were expressed as mean±SD µmol of Trolox equivalents per gram of dry sample from 3 replicated measurements.

### β-Amyloid (Aβ) Aggregation Assay

The effect of LWDH extracts on Aβ aggregation was determined using a thioflavin T (ThT) fluorescence assay as described previously [43]. Briefly, 20 µL of β-amyloid 1–42 (25 µM in 10% DMSO/PBS (1×)) was added to 2 µL sample (25 mg/mL in DMSO) in a 0.2 mL tube and mixed by gentle tapping. Tubes were covered to minimize sample evaporation and incubated in the dark at room temperature with no agitation. The extent of Aβ aggregation was estimated by periodically aliquoting 10 µL of Aβ1–42 in a 96-well plate. To each sample, 200 µL of 10 µM ThT in 0.1 M glycine buffer (pH 8.9) was added and the plate was read on a microplate reader (Varioskan, Thermo, USA) for fluorescence intensity at excitation of 450 nm and emission of 482 nm. All ThT fluorescence experiments were performed in triplicate and data expressed as mean ± SD (N = 3).

### *Caenorhabditis elegans* Strain and Maintenance

The wild-type *C. elegans* strain N2 (Bristol) and transgenic worms, TJ375 (hsp16.2::gfp), CF1553, CL4176 (smg-1ts [myo-3/Aβ1–42 long 3'-untranslated region (UTR)]) and CL2006 were purchased from Caenorhabditis Genetics Center (University of Minnesota, Minneapolis, MN). All *C.elegans* strains were maintained at 20°C except strain CL4176 which was maintained at 16°C on solid nematode growth medium (NGM) seeded with live *E. coli* (OP50) as a food source.

### LWDH Treatment to *C. elegans*

Stock solutions (100 mg/mL) of LWDH extracts were prepared in methanol (LWDH-EE) or water (LWDH-WE). The extracts were added to the nematode growth medium (NGM) to a final concentration of 0.1–2.0 mg/mL of NGM using 0.05–0.1% methanol, just before plating. Gentamycin was added to the NGM at a concentration of 30 µg/mL to inhibit microbial contamination. The *E. coli* OP50 was spread on the NGM as food for *C. elegans*. Plain NGM or MeOH added to the NGM served as controls. The extract of *Ginkgo biloba* (EGb761) (1 mg/mL NGM) and in some experiments, quercetin (200 µg/mL NGM) was used as positive control.

### Bioassays for β-amyloid-induced Paralysis

To determine if LWDH extracts suppress or delay the onset of β-amyloid induced progressive paralysis in CL4176 expressing muscle-specific Aβ 1–42 [44], freshly laid eggs were transferred on to NGM containing LWDH extracts (water-LWDH-WE; ethanol-LWDH-EE) or controls and incubated for 36 h at 16°C. To initiate the amyloid-induced paralysis, the worms were up shifted from 16°C to 23°C. The scoring was performed at an hourly interval typically after 25 h at 23°C. The worms were scored as “paralyzed” based on either the failure of the worms to move their body with touch of a platinum loop, or the formation of a halo on bacterial lawn indicating a paralyzed condition. Each experiment was performed using at least 90 worms. The data represents mean of three different experiments (N = 270).

To determine the effect of LWDH-EE on deposition of amyloid plaques in *C. elegans*, we used transgenic CL2006 nematodes. Synchronous eggs (60–80) were transferred to NGM plates with or without LWDH-EE and incubated at 16°C for 3 days. The worms at late L4 stage (just before adulthood) were shifted into a 20°C environment for further development. Samples were harvested at different times (4 and 8 days after adulthood) and fixed overnight in 4% paraformaldehyde in PBS pH 7.4 at 4°C. The worms were then permeabilized by incubation for 24 h at 37°C in 5% β-mercaptoethanol in 125 mM Tris, pH 7.4 containing 1% Triton X-100. Samples were washed 2–3 times with PBS-T, then mounted on glass slide and stained with 0.125% thioflavin-T in 50% ethanol for 2 min. The nematodes were destained with sequential washes with ethanol (50%, 75% and 90%) and observed under a fluorescent microscope (Olympus, Tokyo, Japan) for the presence of amyloid plaques in the head region. The experiment was repeated 2 times and the data expressed as mean±SE (N = 30) for each group.

### Western Blotting of Aβ Species

Worms were harvested in ddH_2_O containing protease inhibitor cocktail (1×, Sigma), flash frozen in liquid nitrogen and stored at −80°C. Worms were boiled at 105°C for 10 min in a lysis buffer (62 mM Tris-HCl pH 6.8, 2% SDS (w/v), 10% glycerol (v/v), 4% β-mercaptoethanol (v/v) and 1X protease inhibitor cocktail), and then cooled on ice and centrifuged for 5 min at 14,000 g at 4°C. The protein in the supernatant was quantified using Bradford reagent (Biorad). Fifteen µg of protein was denatured prior to electrophoresis by boiling for 5 min in denaturation buffer (62 mM Tris-HCl pH 6.8, 2% SDS (w/v), 10% glycerol (v/v), 4% β-mercaptoethanol (v/v) and 0.0005% bromophenol blue (w/v)). Samples were run at 140 V on 16% SDS BIS-Tris gel using Tris-glycine SDS running buffer (Biorad), for approximately 90 min. Ten to 175 kDa protein markers (Bioshop, Burlington Canada) were used as size references. The gel was transferred to 0.45 µM PVDF membrane (Immobilon P, Millipore) using 20% methanol TG buffer (Biorad), using 20 V for 120 min.

Blots were blocked in TBS-Tween +5% milk (100 mM Tris- 7.5, 150 mM NaCl, 0.1% Tween-20 (v/v)). Amyloid protein species were detected with 6E10 (Covance) at 1∶750 dilution; secondary anti-mouse IgG alkaline phosphatase conjugate (Sigma). Secondary alkaline phosphatase conjugate were developed using SigmaFast™ BCIP®/NBT tablets (Sigma) and the reaction stopped by addition of 20 mM EDTA (pH 8.5). Mean densities of β-amyloid bands were analyzed using Image-J software (National Institutes of Health, USA) on air dried blots. Equal loading was determined by replicate non-transferred Coomassie Blue stained, SDS-PAGE gel. The data is expressed as mean of 5 biological replicates using 4 and 20 kD bands.

### *In vivo* Measurement of Reactive Oxygen Species (ROS) in *C. elegans*

Intracellular ROS was measured in treated and control *C. elegans* strain CL4176 using 2,7-dichlorofluorescein diacetate (H2DCF-DA) following a previously described method [26], [30] with modifications. Briefly, freshly laid eggs (60–65 eggs per plate) were transferred to control or LWDH-EE (0.5 mg/ml) amended NGM plates and incubated for 36 h at 16°C. To initiate amyloid induced progressive paralysis, the worms were shifted to an incubator set at 23°C. The worms were harvested at 30 h after temperature shift using 500 µL of phosphate-buffered saline (PBS), washed twice with PBS to remove *E. coli* (OP-50) cells and transferred into 96-well plate (Costar) in a 200 µL volume of PBS containing Tween 20 (0.01%) and H2DCF-DA (final concentration 50 µM in PBS). The fluorescence was quantified in a Synergy HT microplate fluorescence reader (Bio-Tek Instruments, Winooski, VT) for 6 h at 37°C using the excitation at 485 nm and emission at 530 nm. Data represent mean±SE of three independent experiments expressed as percent fluorescence relative to MeOH control.

### Fluorescence Microscopy of Reporter Gene Expression in LWDH-EE Treated *C. elegans*

To study the effect of LWDH extracts on HSP16.2 and SOD-3, *C. elegans* strains TJ375 (hsp-16.2::GFP) and CF1553 (sod3::GFP) were used. Synchronized TJ375 eggs were transferred on to NGM plates containing the extracts and incubated for 2 days at 20°C. For inducing heat shock response, L4 worms were shifted to a temperature controlled incubator set at 35°C for 2 h followed by recovery at 20°C before taking pictures at 24, 48 and 72 h. For SOD-3 response, pretreated L4 worms (CF1553) were transferred to new NGM plates containing 100 µM juglone and incubated overnight before analyzed with fluorescence microscopy. For quantification, the worms were anesthetized with sodium azide (10 mM) on agarose pad on a glass slide and the fluorescence was viewed under a microscope (Olympus, Japan) with excitations at 488 nm and emissions at 500–530 nm. The fluorescence intensity was analyzed using Image-J software. Each experiment was repeated twice and 15 worms per group were used in each experiment. Data represent mean±SE (N = 30) of two biological experiments.

### Expression Analysis of Stress Induced Genes in *C. elegans*

To relate the phenotypic and biochemical response of the LWDH-EE treated worms with corresponding genes, the transcript of β-amyloid transgene (*amy-1*), superoxide dismutase 3 (*sod3*) and small heat shock protein (*hsp16.2*) were examined. The transgenic *C. elegans* strain CL4176 was treated with LWDH extracts as described in the earlier section. The worms were sampled at 10 and 15 h after temperature upshift. The worms (80–100) were transferred directly into TRIzol Reagent (100 µL) (Invitrogen Life Technologies) and flash frozen in liquid nitrogen. For RNA extraction, 70 µL chloroform was added to TRIzol with worms, mixed well and centrifuged at 10,000 rpm for 10 min. The supernatant was mixed with 70 µL ethanol (70%) and loaded directly onto RNeasy spin columns (Qiagen, Canada) to precipitate RNA according to the manufacturer's protocol. Total RNA was reverse transcribed with 0.5 µg of DNase digested total-RNA using Quantiscript reverse transcriptase (Qiagen, Canada). The induction of β-amyloid gene *amy-1* (forward, 5′-CCGACATGACTCAGGATATGAAGT-3′; reverse, 5′-CACCATGAGTCCAATGATTGCA-3′), small heat shock protein *hsp16.2* (forward, 5′-ACG CCA ATT TGC TCC AGT CT-3′; reverse, 5′-GAT GGC AAA CTT TTG ATC ATT GTT A-3); and *sod-3* (forward, 5′-AGCATCATGCCACCTACGTGA-3′ reverse, 5′-CACCACCATTGAATTTCAGCG-3′) were determined by quantitative Real-Time PCR performed on StepOne™ Real-Time PCR System (Applied Biosystems) using SYBR green reagent (Roche Diagnostics, Mississauga, ON, Canada). The gene *ama-1* (forward, 5′-CTGACCCAAAGAACACGGTGA-3′ reverse, 5′-TCCAATTCGATCCGAAGAAGC-3′) was used as the internal control. Data were analyzed from two independent runs and expressed as mean±SE.

### Juglone Induced Stress in Wild Type N2

Wild type N2 worms were used to determine if LWDH-EE imparts tolerance to juglone induced oxidative stress. Adult N2 worms were allowed to lay eggs for 3 h on NGM plates. Freshly laid eggs were transferred to treatment plates containing LWDH-EE (0.5 mg/ml) or the MeOH control and allowed to develop up to late L4 stage (approximately 2.5 days) at 20°C. *Gingko biloba* extract (EGb761) served as the positive control. The pretreated L4 worms (30 worms/plate, 90 worms per group) were then transferred to NGM plates supplemented with juglone (300 µM). Survival of the worms was observed at hourly intervals until all the worms died in each of the treatment plates. The experiment was repeated two times and data represents mean±SE (N = 180) for each group.

### Statistical Analysis

JMP IN software was used for statistical analyses. Comparison between treatments was done with unpaired Student’s *t* test. For paralysis assays, a two-way ANOVA was used to determine significance. Differences at the p<0.05 level were considered significant and marked with an asterisk (*).

## Results

### Identification and Quantification of Main Components in LWDH Extracts

As a classic TCM formula, LWDH has been well documented in TCM literature for improving cognition and the commercial products are regulated and standardized in China. A number of bioactive components are reported in the LWDH formula. However, the composition of products based on this complex formula may still vary to certain degree from one manufacturer to another. HPLC-based methods for quality control and component analysis have been applied to LWDH, and indeed, minor variations were shown to be evident [45]. We first determined the main bioactive components in LWDH extracts used in our study with ^1^H NMR and HPLC-MS analysis. In proton NMR, LWDH-WE and LWDH-EE are similar in terms of compound classes presented. However, higher sugar signals and lower levels of phenolics and other components were found in LWDH-WE, as compared to LWDH-EE (Figure S1). In HPLC analysis, C-18 column resolved six less polar components and their identities were revealed to be gallic acid, 5-hydroxymethyl furfural, sweroside, loganin, paeoniforin, and paeonol, based on available reference compounds and MS data (Figure S2 and Table S1). For high polarity components, a HILIC HPLC column was used generating the identity of loganin, gallic acid, glycosides of 5-hyrdoxymethyl furfural and gallic acid, monosaccharide monomers, dimers, and trimers as the main components based on ELSD and MS information (Figure S3 and Table S2). Additional monosaccharide dimers and trimers were found in LWDH-WE, as also indicated in the NMR spectra. The main components identified in this study are in general agreement with previously published reports [45]–[47].

Phenolic compounds such as gallic acid and paeonol in LWDH may contribute to its beneficial effects, so we quantified the total phenolics in LWDH extracts. By using Folin-Ciocalteu colorimetric method, the total phenolic contents of LWDH-WE and LWDH-EE extracts were determined to be 14.97±0.36 and 60.43±3.56 mg/g gallic acid equivalent, respectively. The results showed that ethanol extract contained higher concentration of phenolic components than the water extract, as revealed by NMR and HPLC analysis.

We further quantified the contents of 6 main components identified in LWDH extracts using HPLC-DAD method (Figure S4). 5-Hydroxymethyl furfural content was determined to be 18.788±0.071 and 2.065±0.004 mg/g in LWDH-EE and LWDH-WE, respectively, the highest component measured for these extracts. Two phenolic compounds, gallic acid and paeonol, were 5.144±0.168 and 4.675±0.004 mg/g in LWDH-EE, while gallic acid was measured to be 0.776±0.003 mg/g in LWDH-WE, much lower than that of LWDH-EE. The other phenolic component, paeonol, however, was even not-detectable in LWDH-WE. Other components, including two iridoids loganin and sweroside, and paeoniforin were measured at 6.167±0.025, 0.787±0.030, and 3.676±0.210 in LWDH-EE, and 0.850±0.006, 0.104±0.004, and 0.483±0.013 mg/g in LWDH-WE, respectively. The ethanol extract not only showed to have higher content of phenolic compounds, but also contained specific compound such as paeonol that was not present in water extract. Significantly higher levels of other components in LWDH-EE may also be responsible for the observed differences in bioactivity between these two extracts.

### DPPH Radical Scavenging Activity and Effect on Aβ Aggregation of LWDH Extracts

We measured the antioxidant activity of LWDH extracts using DPPH radical scavenging assay. DPPH, 1,1-diphenyl-2-picrylhydrazyl, is a stable organic nitrogen radical with maximum UV-vis absorption at 515 nm. When it interacts with antioxidant, the solution color fades and this is monitored by a spectrometer. In our assay, antioxidant standard (+/−)-6-hydroxy-2,5,7,8-tetramethyl-chromane-2-carboxylic acid (Trolox) was used for the measurement. The results revealed that the ethanol extract LWDH-EE showed significantly higher antioxidant activity than the water extract LWDH-WE. The radical scavenging activities of LWDH-WE and LWDH-EE were 102.20±5.52 and 402.76±18.52 µmol Trolox equivalent (TE)/g, respectively. The high DPPH activity of ethanol extract correlated with higher phenolic content of LWDH-EE.

Aβ aggregation, fibril formation and deposition are believed to cause neurotoxicity and eventually the onset of neurodegenerative diseases, such as AD [48], [49]. Polyphenols and other types of small molecules are reported to be potential inhibitors of Aβ aggregation [43], [50]–[52], suggesting this to be, at least in part, mechanisms of action for prevention and treatment of AD. By using the ThT fluorescence method and curcumin as a positive control [53], [54], we determined the effect of LWDH extracts on Aβ aggregation. However, LWDH extracts did not affect Aβ aggregation, suggesting that LWDH extracts appears not to modify or intervene in the Aβ aggregation process, especially *in vitro*.

### Aβ-induced Paralysis in the Transgenic *C. elegans* is Alleviated by LWDH Extracts

**Figure 1:**
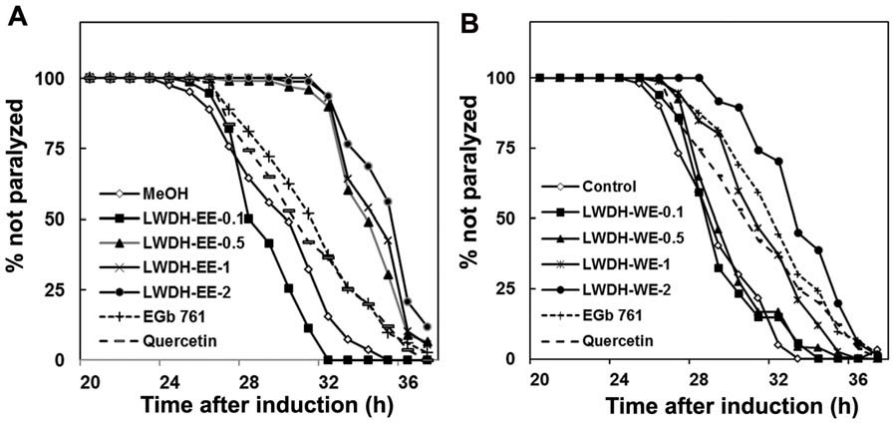
LWDH delayed β-amyloid induced paralysis in transgenic *C. elegans*, strain CL4176. Time course of Aβ-induced paralysis in the transgenic CL4176 strain treated with LWDH ethanol (A), and water (B) extract. Egb761 and quercetin served as positive controls whereas untreated NGM or MeOH +NGM were used as controls. *C. elegans* paralysis phenotype was monitored 20 h following temperature upshift to 23°C and expressed as percent survival of the worms drawn based on three individual experiments with 90 worms in each group (N = 270). The significances of differences in paralysis curves were p<0.05 versus the control.

We used *C. elegans* strain CL4176 expressing Aβ1–42 [55] to determine the effects of LWDH on Aβ-induced toxicity in the whole animal. The results showed that LWDH-EE was highly effective as compared to LWDH-WE in protecting *C. elegans* against β-amyloid induced toxicity (Figure 1). *C. elegans* treated with ethanol extract of LWDH >0.1 mg/mL delayed the onset of paralysis compared to control (MeOH). This effect was not significantly different between 0.5–2 mg/mL concentrations of LWDW-EE tested (p>0.05) (Figure 1A). LWDH-EE treated worms showed paralysis after 30 h while the onset of paralysis started at 25 h in the MeOH control and more than 50% of worms were paralyzed by this time (Figure 1A). In contrast, LWDH-WE effect was not as strong as LWDH-EE where the paralysis phenotype appeared by 27 h after temperature induction. The higher dosage (1–2 mg/mL) of LWDH-WE showed an increasing effect in delaying the paralysis phenotype than NGM control but this effect was not as strong as LWDH-EE (0.5–2 mg/mL) that showed higher efficiency (p<0.05) versus the control in delaying paralysis. When compared with positive control, EGb761 (1 mg/mL), the effect of LWDH-WE (1–2 mg/mL) was similar to the Ginkgo extract (p>0.05) (Figures 1A & 1B). Quercetin (200 µg/mL) a potent antioxidant, did not negate paralysis. These observations suggest that LWDH and more specifically LWDH-EE may have the potential to protect against β-amyloid induced toxicity.

**Figure 2:**
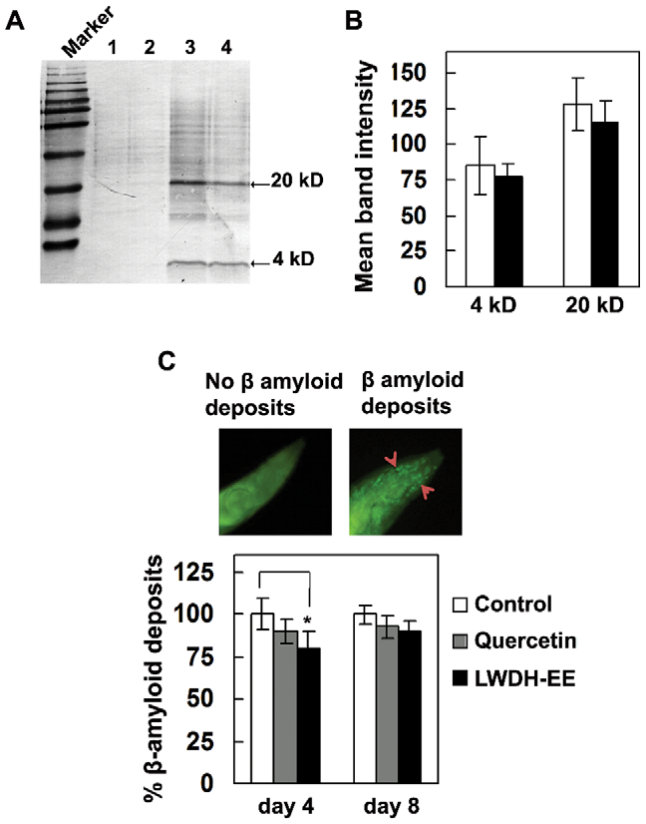
LDWH reduced β-amyloid species in *C. elegans*. A. Western blot of Aβ species in the transgenic *C. elegans* CL4176 with and without LWDH-EE treatment. The CL4176 proteins were loaded on 16% acrylamide gel and immunoblotted with an anti-Aβ antibody (6E10). Lanes 1 and 2 were loaded with CL4176 proteins from 16°C whereas lane 3 (MeOH control) and lane 4 (LWDH-EE) were loaded with protein extracted from worms incubated in permissive temperature. The blot represents five independent experiments. B. Quantification of Aβ species (4 and 20 kD) using ImageJ software. Data are expressed as mean density of two indicated bands±SE based from five independent experiments. C. Aβ species in CL2006 determined by Thioflavin-T stain. CL2006 worms were treated with LWDH-EE and stained with ThT and viewed for amyloid aggregation in the head region anterior to the pharyngeal bulb. All ThT fluorescence experiments were performed two times. Data were obtained from two biological experiments with 15 worms in each group (N = 30) and expressed as β-amyloid deposits relative to control worms. Error bars represent SEM. *p<0.05.

To determine if the effect of LWDH on delayed onset of paralysis was associated with a decrease in Aβ accumulation in transgenic CL4176, we performed Western blot analysis of LWDH-EE treated worms sampled at 25 h after temperature upshift (Figure 2A). The visual observation of immunoblot developed with anti-Aβ monoclonal antibody 6E10 did not show significant difference in monomeric or oligomeric Aβ species. The quantitative analysis of Aβ bands with ImageJ showed a small reduction in Aβ proteins (4 and 20 kD) but this difference was not significant (Figure 2B).

When we tested the effectiveness of LWDH-EE on *C. elegans* strain CL2006, which constitutively express Aβ, a marginal delay in paralysis was observed (data not shown). Further, β-amyloid deposits were detected in the head region of control and treated worms (Figure 2C). ImageJ analysis showed that LWDH-EE reduced β-amyloid deposits in the worms by day 4 after adulthood, the effect of LWDH-EE however diminished with time as the difference was no longer significant by the 8^th^ day. Quercetin used in this experiment as positive control also failed to suppress β-amyloid deposition. These results seem to indicate that the protective effect of LWDH against β-amyloid induced toxicity may be, in part, due to the delayed Aβ accumulation and deposition in the worms that contributes to delayed onset of paralysis phenotype.

### Ethanol Extract of LWDH Reduces Oxidative Stress in *C. elegans*

**Figure 3:**
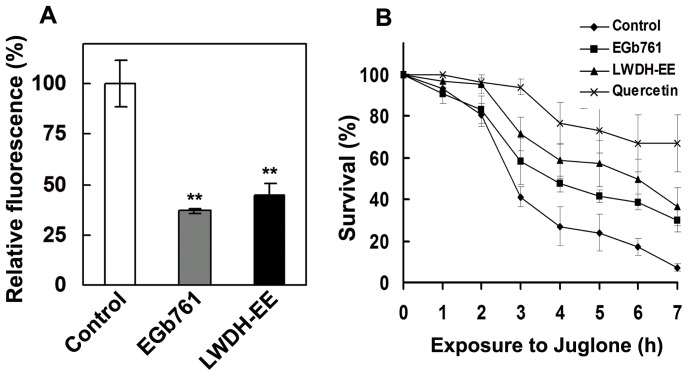
Effect of LWDH on reactive oxygen species (ROS) and Juglone induced oxidative stress tolerance in *C. elegans*. A. ROS were measured in LWDH-EE, Egb761 and untreated CL4176 worms using 2,7-dichlorofluorescein diacetate. Results are expressed as DCF (2,7-dichlorofluorescein diacetate) fluorescence relative to the untreated control. EGb761 (1 mg/mL) served as positive control. Data represent mean+SE, **p<0.001. B. Survival assay of N2 under juglone (300 µg/mL) generated oxidative stress. Survival was scored at 1 h interval until all the worms died. All survival curves are based on two independent experiments with 90 worms in each experiment (N = 180, p<0.05). EGb761 (1 mg/mL), quercetin (200 µg/mL) and plain NGM served as controls.

LWDH extracts were shown to be potent in scavenging DPPH radical *in vitro*. To determine whether LWDH extract has effect in reducing reactive oxygen species (ROS) in *C. elegans*, we investigated the *in vivo* ROS activity in LWDH-EE treated and control worms using transgenic strain, CL4176. LWDH-EE reduced ROS in the worms as compared to control. This was evident by a highly significant reduction (p<0.001) in mean fluorescence detected during 6 h period (Figure 3A). The results showed that LWDH-EE suppressed ROS by more than 50%. This response was similar to the effect of a well known antioxidant EGb 761. These results suggest that the delayed onset of Aβ related paralysis phenotype in CL4176 worms with LWDH treatment might be, in part, due to the ROS suppressing properties of the LWDH extract.

We further observed that the LWDH-EE also protected wild type N2 against oxidative stress generated by juglone. The worms pre-incubated in LWDH-EE (0.5 mg/mL) were significantly protected against juglone (300 µM) induced oxidative stress (Figure 3B). The mean survival of the worms was significantly higher (40%) (N = 180, p<0.05) with LWDH-EE at 6 h after the stress compared to the control (Figure 3B). The T50 (time to 50% mortality) was observed at 3 h for control and 5.8 h for the LWDH-EE treated worms (p<0.05). The survival of the worms with quercetin treatment was significantly higher than LWDH-EE and EGb761 at 7 h. Although the survival of the worms with LWDH-EE treatment during juglone induced oxidative stress was higher than that of EGb761, this difference was, however, not significant (p>0.05).

### Gene Expression Analysis of LWDH-EE Treated *C. elegans*

**Figure 4:**
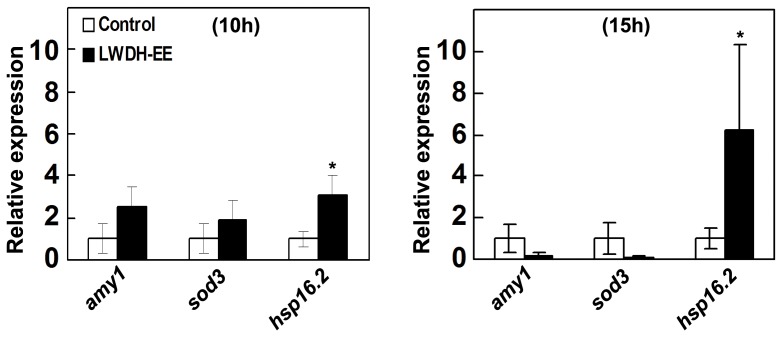
Gene expression in transgenic *C. elegans*, strain CL4176. Quantitative real-time PCR results of LWDH-EE treatment on the expression of the *amy1, sod3* and *hsp16.2* in *C. elegans* strain CL4176. Error bars indicate ±SE. *C. elegans* were raised on NGM or NGM containing LWDH-EE at 16°C and then shifted after 36 h to 23°C. The worms (∼80–100) were harvested at 20 h after temperature upshift for RNA extraction. The relative gene expression was determined with 2^−ΔΔCt^ method. The gene *ama-1* was used as the internal control. Data were average from two independent runs (*p<0.05).

To relate the effect of LWDH-EE on the molecular response in *C. elegans*, the transcript levels of three genes, *amy-1*, *sod3* and *hsp16.2* were studied in transgenic *C. elegans* strain CL4176 after temperature upshift. Quantitative Real-Time PCR revealed differential expression of these genes after temperature upshift (Figure 4). The results showed that the transcripts of all three genes (*amy-1*, *sod3* and *hsp16.2*) were initially higher in LWDH-EE treatment than control at 10 h after temperature upshift. However, *amy-1* and *sod-3* transcript abundance was less in LWDH treated worms as compared to the control at 15 h after the temperature upshift. Interestingly, the *hsp16.2* transcript, which was higher at 10 h, further increased to more than six fold (p<0.05) with LWDH-EE treatment as compared to the control.

### LWDH-EE Increases hsp-16.2::GFP Expression in *C. elegans*

**Figure 5:**
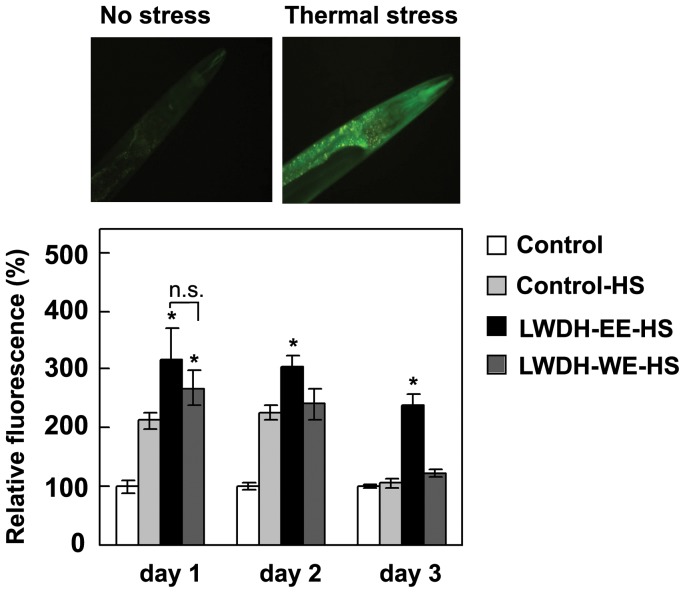
Effect of LWDH on the expression of hsp16.2::GFP in *C. elegans*. GFP fluorescence images of the *C. elegans* strain TJ375 expressing hsp16.2::GFP were examined under an epifluorescence microscope (Inset). For fluorescence quantification, images at 24, 48 and 72 h were analyzed using ImageJ software (National Institutes of Health, USA). Data are expressed as HSP-16.2::GFP intensity (mean±SE) relative to control was obtained from two independent experiments with at least 15 worms in each experimental group (N = 30, *p<0.05).

LWDH induced stress response in transgenic *C. elegans* strain TJ375 carrying hsp16.2::GFP reporter was determined after 2 h thermal stress (35°C) and overnight recovery at 20°C. The GFP fluorescence quantified in the head region of the worms showed a remarkably higher (p<0.05) hsp-16.2::GFP expression after heat shock with LWDH-EE compared to the LWDH-WE or untreated control worms (Figure 5). The LWDH-EE-specific effect remained evident even at day 3 post thermal stress with almost twice the expression (p<0.05) than other treatments. These results are in agreement with the gene expression results from *C. elegans* strain CL4176 described above, and suggests that LWDH-EE treated worms might be protected from Aβ toxicity due to an up regulation of HSP16.2.

### LWDH-EE Affects the Expression of sod3::GFP in *C. elegans*

**Figure 6:**
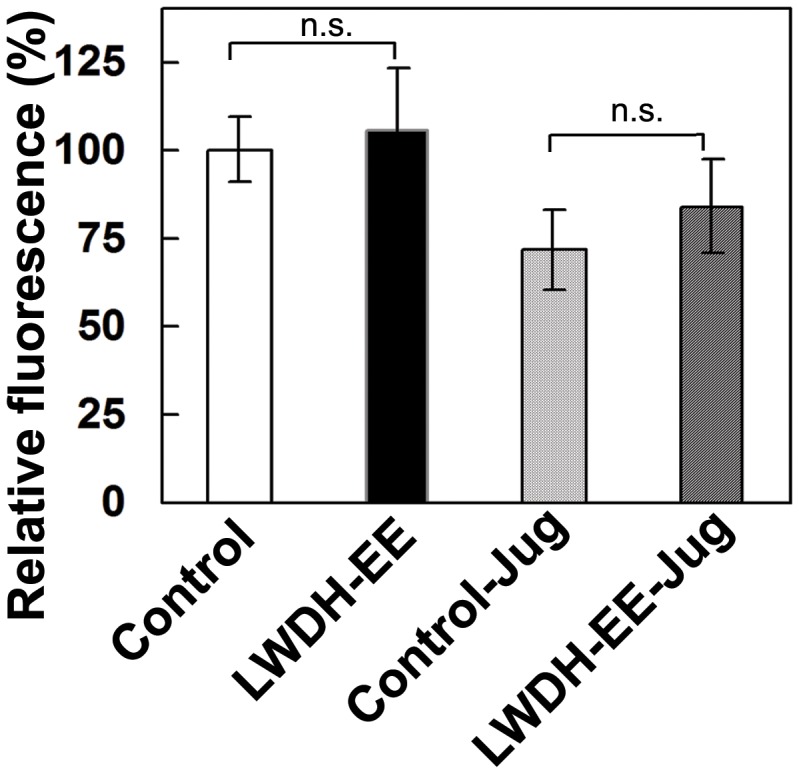
Effect of LWDH on the expression of sod3::GFP in *C. elegans*. To determine role of SOD-3 in LWDH induced response of *C. elegans*, L4 worms of CF1553 previously treated from egg stage with LWDH-EE were exposed to 100 µM juglone overnight and then observed for the fluorescence. The fluorescence intensity was analyzed using Image-J software (National Institutes of Health, USA). The images were quantified based on GFP intensity (mean±SE) in CF1553 from two independent experiments with 15 worms in each experiment (N = 30, *p<0.05).

The molecular response in *C. elegans* treated with LWDH-EE under oxidative stress was further studied using transgenic worms (CF1553) expressing sod3::GFP. The control or LWDH-EE treated L4 worms were subjected to overnight exposure to 100 µM juglone. Interestingly, relative fluorescence of sod3::GFP in different biological replicates were only 5–10% higher in LWDH-EE treated worms at normal conditions (no juglone stress) compared to control worms the difference was however not significant (Figure 6). Similar pattern was observed with juglone treatment as the increase in fluorescence intensity was not significant in LWDH-EE treated worms compared to control worms (Figure 6). It appears that the tolerance to juglone induced stress in the LWDH-EE treated worms might involve additional molecular responses along with SOD-3.

## Discussion

Alzheimer's disease (AD) is a neurodegenerative disease associated with ageing [56]. Besides extensive pre-clinical and clinical research on pathology and etiology of this disorder, other than symptomatic treatment strategies for treating this debilitating disease are used, the cause and a cure for AD have yet to be fully elucidated and achieved. The future success of the treatment and management of AD is therefore highly dependent on early detection and treatment with drugs that block, mitigate, slow, or suppress any of the causative and/or putative disease-associated co-factors and delay the onset of AD. Traditional Chinese medicine, along with other natural product-based therapies have been used in preventing or treating age-related deterioration in cognitive or memory performance [57]–[59], and a number of studies have been conducted to examine their potential benefits in AD.

Liuwei Dihuang (LWDH), a classic six-herb TCM formula, has been used for over a thousand years to improve or restore declined functions related to aging process and geriatric diseases, such as impaired mobility, vision, hearing, cognition and memory [1]. Several studies showed that LWDH can modulate neuronal and synaptic function that contributes to the cognitive enhancement through improved functional neurotransmission between neurons [6], [9]. However, it was not clear if β-amyloid toxicity was alleviated in *C. elegans* expressing β-amyloid after LWDH treatment.

We examined if LWDH has *in vitro* and/or *in vivo* effect on β-amyloid based on experimental evidences from Aβ aggregation, expression and/or deposition. Although, LWDH extracts did not reduce aggregation of amyloid protein *in vitro*, LWDH extracts were found to be effective *in vivo* and we observed a delayed onset of β-amyloid induced paralysis in *C. elegans* strain CL4176 expressing Aβ peptide [21] (Figure 1). When examined for toxic amyloid plaques in *C. elegans* strain CL2006, β-amyloid deposits were initially low with quercetin and LWDH-EE treatments (Figure 2), the difference however diminished with age. Gene expression study demonstrated that LWDH suppressed *amy1* in *C. elegans* strain CL4176 suggesting that the treatment did not have a significant effect on the transcription of amy1 gene. Aβ transgene expression has not always correlated with β-amyloid deposition in the worms expressing Aβ1–42 [30], [35], [37]. One possible explanation is the differences in transgenic strains used in the experiments and the time of sampling the worms for analysis. During initial (at 10 h) observations, we also noted upregulation of *amy1* gene in CL4176 with LWDH-EE treatment, however there was repression of *amy1* gene at 15 h after temperature upshift (Figure 4). Results suggest that delayed onset of the paralysis was at least in part due to reduced *amy1* transcription leading to delay in β-amyloid deposition during the temperature upshift. It remains to be determined if LWDH-EE treatment delayed paralysis correlate with differential oligomerization of Aβ species.

To determine potential mechanisms of LWDH activity in the worm, we examined whether it affected the oxidative stress response in *C. elegans*. LWDH possesses strong antioxidant activity [60], [61] that might be one of the contributing factors for the delayed onset of paralysis in the worms. LWDH-EE offered significant protection against oxidative stress in *C. elegans* as evidenced by results of our *in vivo* experiments. Oxidative stress is involved in AD pathogenesis and accumulation of ROS along with the deposition of toxic amyloid species is proposed to exacerbate the condition in AD patients [62], [63]. The antioxidant-based therapies have been suggested as a potential avenue to mitigate AD as has been demonstrated using an AD mouse model [64], [65]. In *C. elegans*, oxidative stress strongly correlates with Aβ toxicity [66] and it is well known that a number of natural products reduce reactive oxygen species which are shown to be neuroprotective [31]. Our results support the hypothesis that ROS reducing activity of LWDH might be one of the mechanisms by which it protected *C. elegans* against Aβ toxicity. Strong ROS scavenging activity did not, however, reduce Aβ accumulation in LWDH treated worms suggesting that formation of toxic pre-fibrillar Aβ species [66] could have been reduced in LWDH treated CL4176 worms.

Small heat shock proteins (HSPs) are collection of low molecular weight polypeptides with chaperone- like activity that increase survival of *C. elegans* under stress and are induced with Aβ expression [44]. Since heat shock response in *C. elegans* is a neuronally-controlled behavior [67], induction of HSP-16 by Aβ is suggested to be a protective response to the abnormal accumulation of toxic proteins [68]. Previous reports showed that *in vivo* β-amyloid peptide toxicity was suppressed by over expression of HSP-16.2 in *C. elegans* [68]. The LWDH treated worms showed significantly higher expression of heat shock proteins as determined through analysis of *hsp16.2* transcript abundance and GFP reporter expression. The GFP expression was elevated even up to 3 days after heat shock suggesting that HSP-16.2 was involved in the protection against Aβ induced toxicity by LWDH. Our results are in close agreement to previous reports and suggest that LWDH might play role in increasing the expression of this small heat shock protein in *C. elegans* which might contribute to suppression of or delayed the onset of paralysis in the treated animals. Mechanistically, this could be possible through enhanced plaque clearance but cellular distribution of Aβ or modulations of multimerization pathways of Aβ that reduce the formation of toxic oligomer species, could also be speculated and worthy of further examination [68].

In summary, LWDH extracts, particularly the ethanol extract (LWDH-EE) alleviates β-amyloid induced paralysis in *C. elegans* through increasing heat shock proteins (HSP16.2), lowering *amy1* and reducing ROS, all of which could have mutually exert the protective mechanisms against β-amyloid toxicity. The finding that β-amyloid induced paralysis was alleviated in *C. elegans* AD model potentially provide more insights into the action of LWDH as a potential neuroprotective agent and warrants further testing to be conducted using higher order experimental models.

## Supporting Information

Figure S1 **^1^H-NMR comparison of LWDH extracts.** A. LWDH-EE; B. LWDH-WE.
(PDF)

Figure S2 **HPLC chromatograms (C-18) of LWDH extracts.** A. LWDH-EE; B. LWDH-WE.
(PDF)

Figure S3 **HPLC chromatograms (HILIC) of LWDH extracts.** A. LWDH-EE; B. LWDH-WE.
(PDF)

Figure S4 **HPLC chromatograms of LWDH extracts in quantitative analysis.** A. Standards mix. 1, gallic acid; 2, 5-hydroxymethyl furfural; 3, sweroside; 4, loganin; 5, paeoniforin; 6, paeonol; B. LWDH-WE; C. LWDH-EE.
(PDF)

Table S1 **MS fragmentation of major compounds in LWDH extracts separated with C18 column.**
(DOC)

Table S2 **MS fragmentation of major compounds in LWDH extracts separated with HILIC column.**
(DOC)
