# Supplementary figures and images for: Liuwei Dihuang (LWDH), a Traditional Chinese Medicinal Formula, Protects against β-Amyloid Toxicity in Transgenic Caenorhabditis elegans
Source: PLoS One. 2012 Aug 30;7(8):e43990. doi: 10.1371/journal.pone.0043990 (PMC3431378; doi:10.1371/journal.pone.0043990)

Figure S1

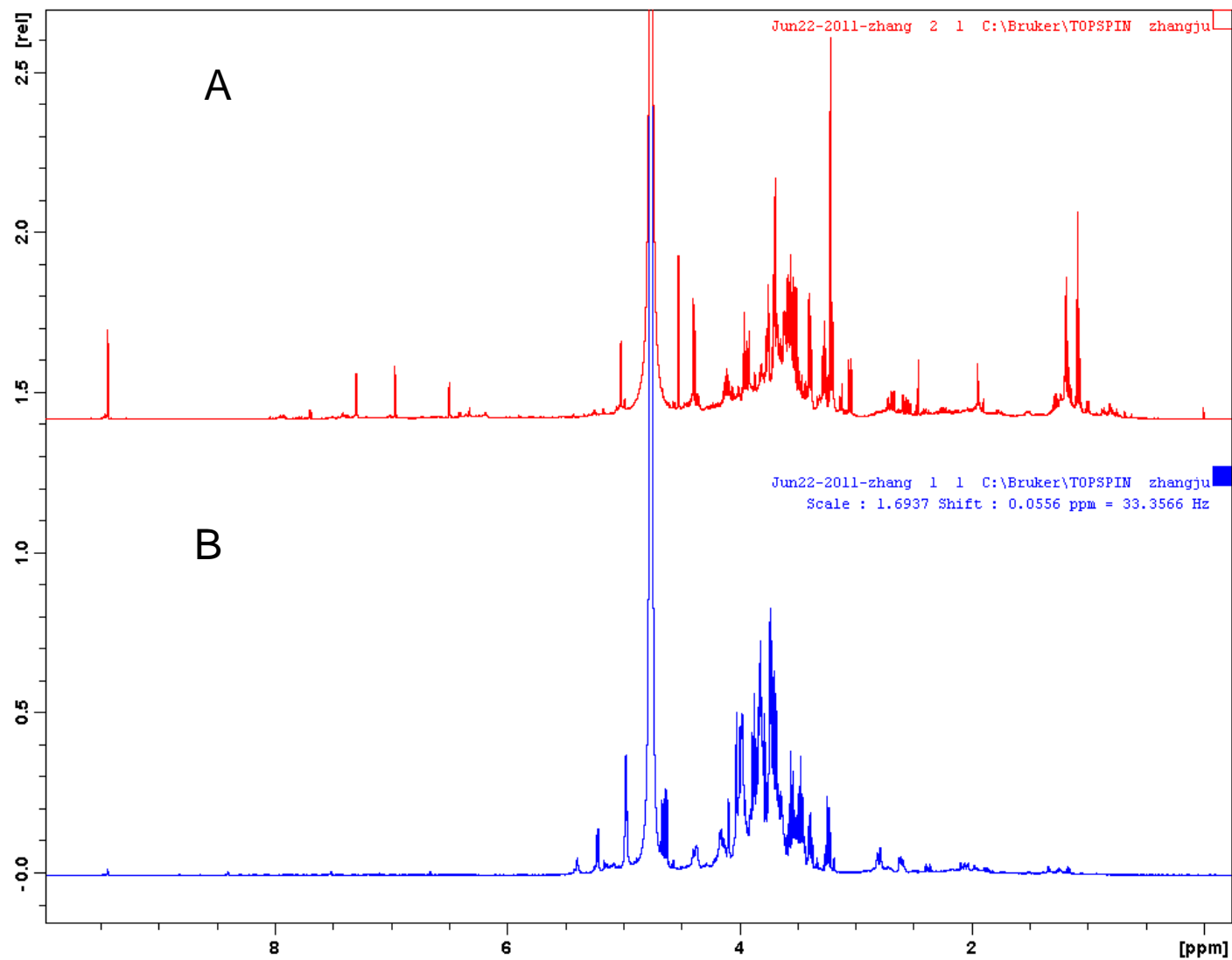

Supplement: Figure S1 — 1H-NMR comparison of LWDH extracts. A. LWDH-EE; B. LWDH-WE. (PDF) [file pone.0043990.s001.pdf]

**Figure S2**

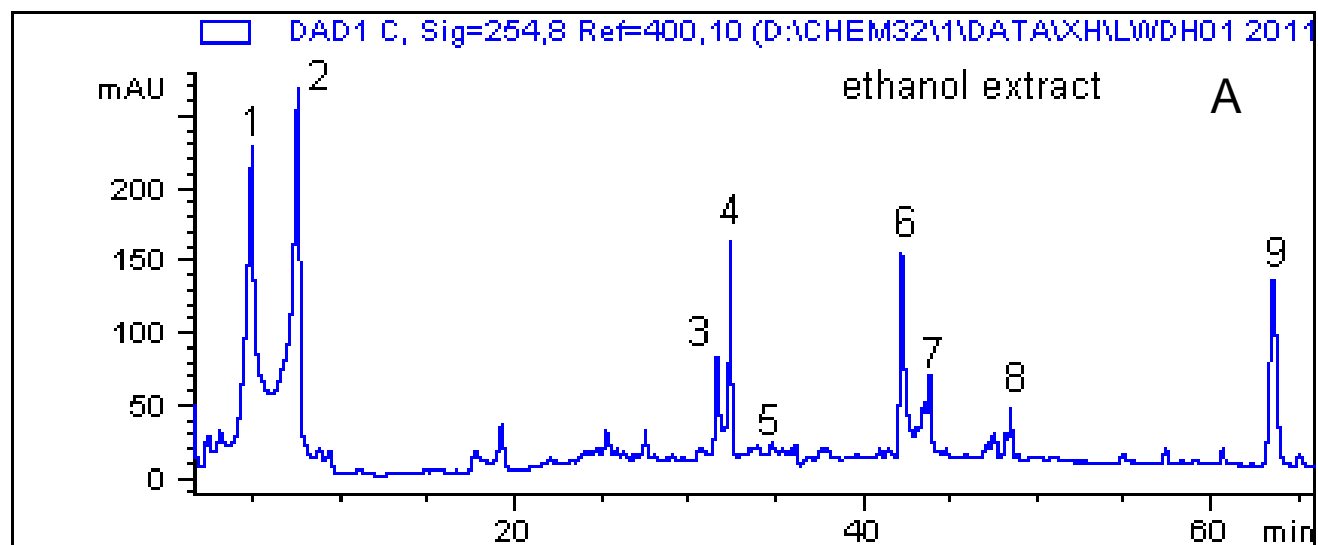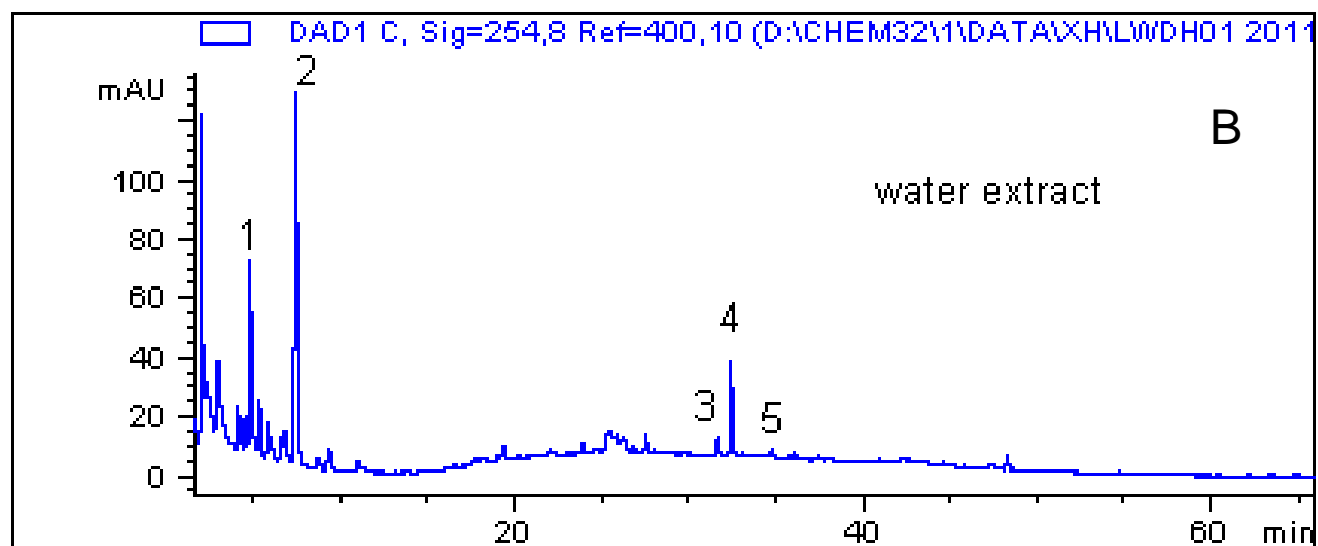

Supplement: Figure S2 — HPLC chromatograms (C-18) of LWDH extracts. A. LWDH-EE; B. LWDH-WE. (PDF) [file pone.0043990.s002.pdf]

**Figure S3**

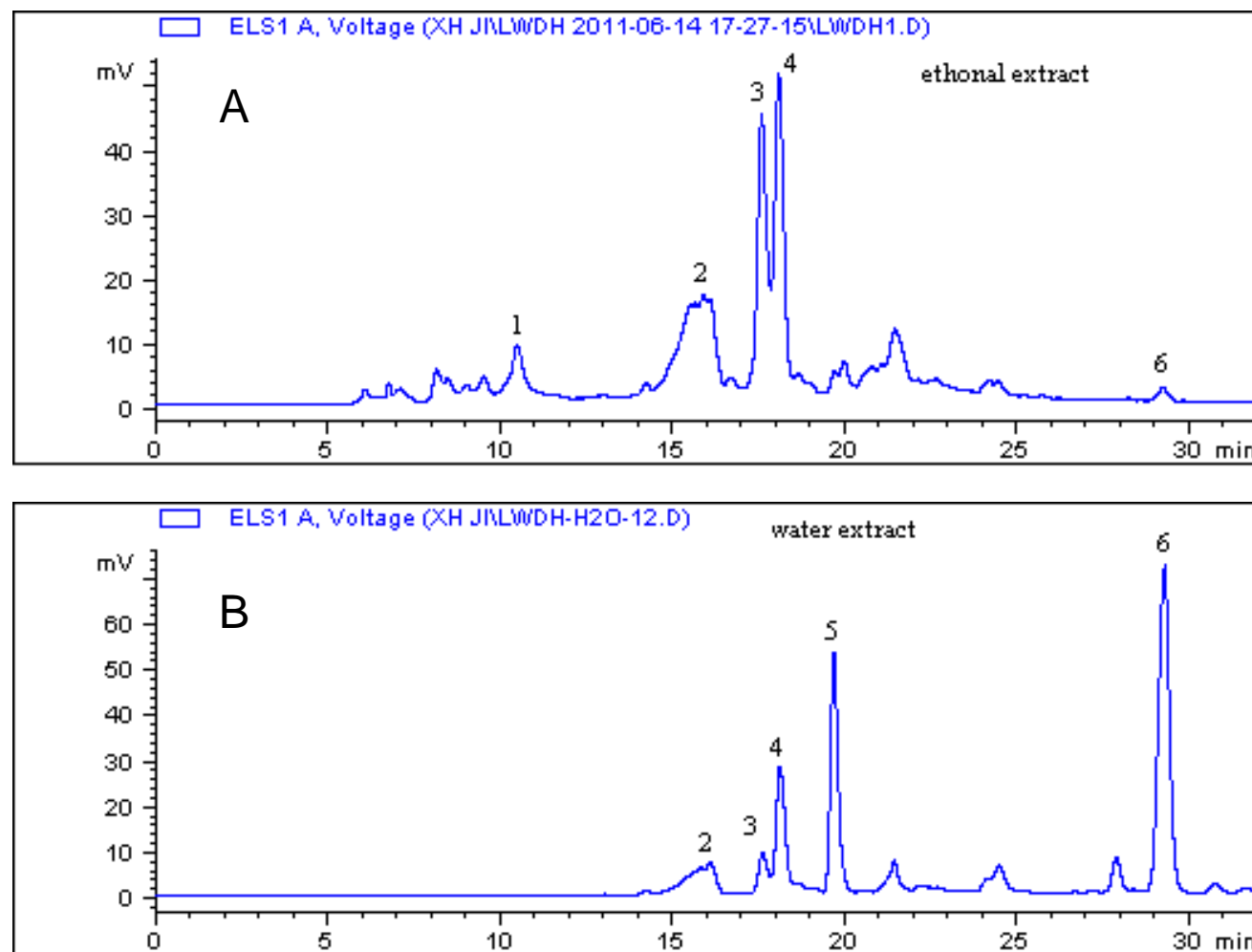

Supplement: Figure S3 — HPLC chromatograms (HILIC) of LWDH extracts. A. LWDH-EE; B. LWDH-WE. (PDF) [file pone.0043990.s003.pdf]

**Figure S4**

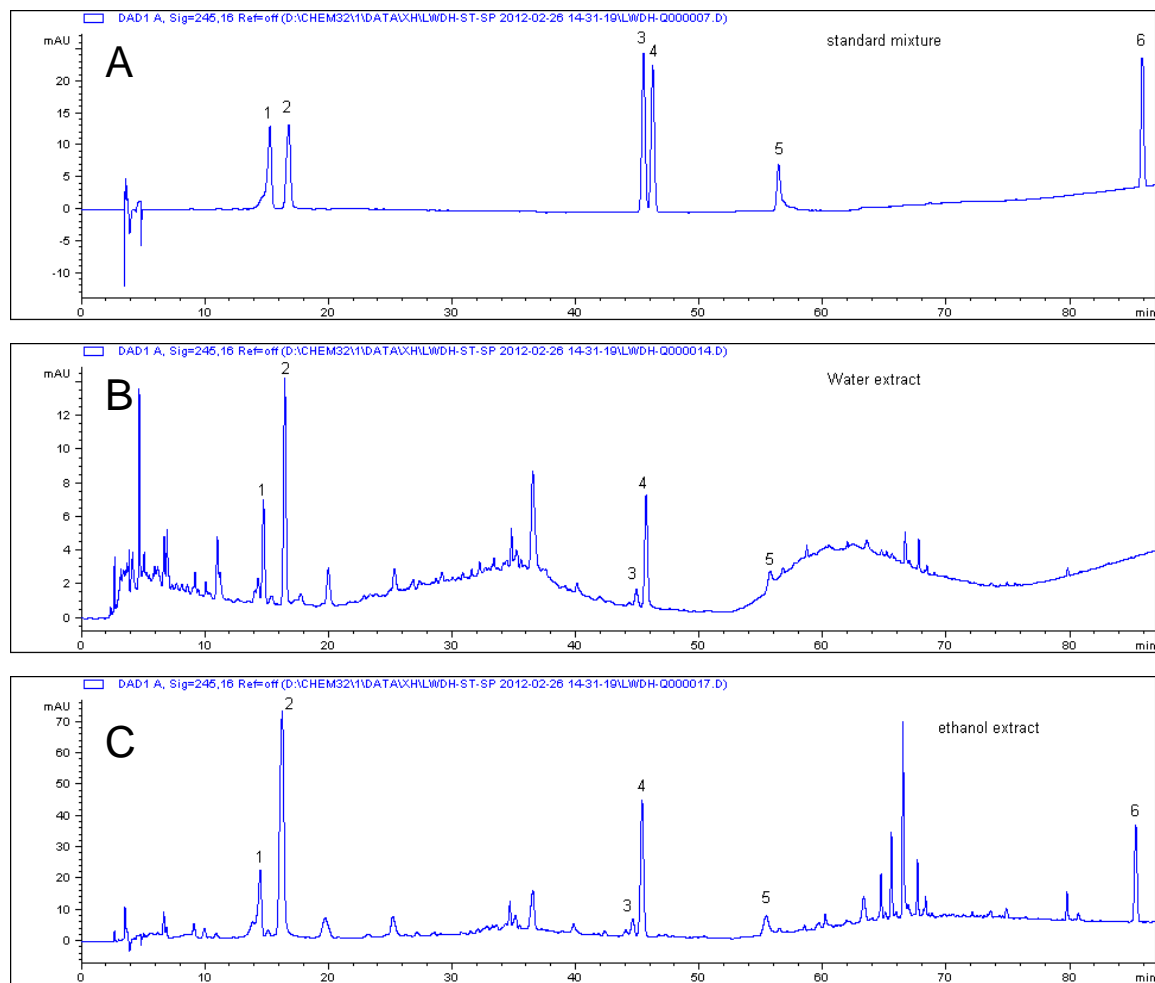

Supplement: Figure S4 — HPLC chromatograms of LWDH extracts in quantitative analysis. A. Standards mix. 1, gallic acid; 2, 5-hydroxymethyl furfural; 3, sweroside; 4, loganin; 5, paeoniforin; 6, paeonol; B. LWDH-WE; C. LWDH-EE. (PDF) [file pone.0043990.s004.pdf]
