# Supplementary material for: Liuwei Dihuang (LWDH), a Traditional Chinese Medicinal Formula, Protects against β-Amyloid Toxicity in Transgenic Caenorhabditis elegans
Source: PLoS One. 2012 Aug 30;7(8):e43990. doi: 10.1371/journal.pone.0043990 (PMC3431378; doi:10.1371/journal.pone.0043990)
Supplement: Table S1 — MS fragmentation of major compounds in LWDH extracts separated with C18 column. (DOC) [file pone.0043990.s005.doc]

**Table S1**

| **Peak No.** | **t_R_ (min)** | **Ions (*m/z*)** | | **M.W.** | **Possible components** |
| --- | --- | --- | --- | --- | --- |
|  |  | **positive ion** | **negative ion** |  |  |
| 1 | 4.8 | - | 169[M-H]^-^, 339[2M-H]^-^ | 170 | gallic acid |
| 2 | 7.4 | 127[M+H]^+^, 109[M+H-H2O]^+^ | - | 126 | 5-hydroxymethyl furfural |
| 3 | 31.5 | 359[M+H]^+^, 197[M+H+H2O-glu]^+^ | 403[M+HCOO]^-^ | 358 | sweroside |
| 4 | 32.3 | 229[M+H+H2O-glu]^+^, 413[M+Na]^+^ | 435[M+HCOO]^-^ | 390 | loganin |
| 5 | 34.7 | 498[M+NH4]^+^ | 525[M+HCOO]^-^ | 480 | paeoniforin |
| 6 | 42.2 | - | - |  | Unknown |
| 7 | 43.8 | 266 | 479 |  | Unknown |
| 8 | 48.4 | 211, 381 | 541, 655 |  | Unknown |
| 9 | 63.5 | 167[M+H]^+^ | - | 166 | paeonol |
