# Supplementary material for: Liuwei Dihuang (LWDH), a Traditional Chinese Medicinal Formula, Protects against β-Amyloid Toxicity in Transgenic Caenorhabditis elegans
Source: PLoS One. 2012 Aug 30;7(8):e43990. doi: 10.1371/journal.pone.0043990 (PMC3431378; doi:10.1371/journal.pone.0043990)
Supplement: Table S2 — MS fragmentation of major compounds in LWDH extracts separated with HILIC column. (DOC) [file pone.0043990.s006.doc]

**Table S2.**

| **Peak No.** | **t_R_ (min)** | **Negative ion (*m/z*)** | **M.W.** | **Possible components** |
| --- | --- | --- | --- | --- |
| 1 | 10.5 | 169[M-H]^-^, 435[M+HCOO]^-^ | 170, 390 mix | loganin and gallic acid |
| 2 | 16 | 179[M-H]^-^, 225[M+HCOO]^-^, 293[M+Cl]^-^, 367 [M+Cl]^-^ | 180, 258, 332mix | Monosacharride, 5-hydroxymethyl furfural with monosacharride and gallic acid with monosacharride |
| 3 | 17.6 | 179[M-H]^-^, 225[M+HCOO]^-^ | 180 | monosacharride |
| 4 | 18.1 | 179[M-H]^-^, 225[M+HCOO]^-^ | 180 | monosacharride |
| 5 | 19.7 | 295[M-H]^-^, 455[M-H]^-^ | 296, 456 mix | di and tri-saccharide |
| 6 | 29.2 | 503[M-H]^-^, 549[M+HCOO]^-^ | 504 | tri-saccharide |
